# Transcriptomic analysis of polysaccharide utilization loci reveals substrate preferences in ruminal generalists *Segatella bryantii* TF1-3 and *Xylanibacter ruminicola* KHP1

**DOI:** 10.1186/s12864-024-10421-z

**Published:** 2024-05-20

**Authors:** Urška Murovec, Tomaž Accetto

**Affiliations:** Department of microbiology, Biotechnical faculty, University of Ljubljana, Groblje 3, 1230 Domžale, Slovenia

**Keywords:** *Segatella*, *Xylanibacter*, *Prevotella*, Transcriptomics, Polysaccharide utilization locus, Rumen

## Abstract

Bacteria of the genera *Xylanibacter* and *Segatella* are among the most dominant groups in the rumen microbiota. They are characterized by the ability to utilize different hemicelluloses and pectin of plant cell-wall as well as plant energy storage polysaccharides. The degradation is possible with the use of cell envelope bound multiprotein apparatuses coded in polysaccharide utilization loci (PULs), which have been shown to be substrate specific. The knowledge of PUL presence in rumen *Xylanibacter* and *Segatella* based on bioinformatic analyses is already established and transcriptomic and genetic approaches confirmed predicted PULs for a limited number of substrates. In this study, we transcriptomically identified additional different PULs in *Xylanibacter ruminicola* KHP1 and *Segatella bryantii* TF1-3. We also identified substrate preferences and found that specific growth rate and extent of growth impacted the choice of substrates preferentially used for degradation. These preferred substrates were used by both strains simultaneously as judged by their PUL upregulation. Lastly, β-glucan and xyloglucan were used by these strains in the absence of bioinformatically and transcriptomically identifiable PUL systems.

**Supplementary Information:**

The online version contains supplementary material available at 10.1186/s12864-024-10421-z.

## Background

Ruminal members of genus *Prevotella* were recently reclassified into genera *Xylanibacter* and *Segatella* [1]*.* The majority of them belong to *Xylanibacter* while *Segatella,* which includes also important human gut species *Segatella copri*, is rarer in the rumen [2]. They seem to be the dominant bacterial component of the rumen microbiome as judged by cultivation, amplicon NGS-based and quantitative transcriptomics studies [3–5]. They are established in the rumen early in life and kept stably for the lifespan of the animal [6]. Their main function appears to be in the breakdown of hemicelluloses and pectin of plant cell-wall as well as plant energy storage polysaccharides [2]. In this, the rumen *Xylanibacter* and *Segatella*, like the vast majority of studied *Bacteroidetes*, rely on polysaccharide utilization loci (PULs), which are inducible clusters of coregulated genes that encode sensing, binding, transport and depolymerization proteins targeted to the outer and inner membrane or periplasm, depending on their exact function [7]. PULs are typically directed at single glycan substrates [8]. The seminal transcriptomic and gene inactivation studies of human gut dwelling *Bacteroides thetaiotaomicron* and *Bacteroides ovatus* identified the targets of their PULs [9, 10] and led to major research efforts which investigated many of these PULs and their products in great genetic and biochemical detail [8]. On the other hand, the delineation of PULs in *Bacteroides* proved very useful for functional annotation of PULs observed in *Xylanibacter* and *Segatella*, which are their close relatives [11]. Distinct complements of PULs and Carbohydrate Acting Enzymes (CAZymes) were found in variety of *Prevotella, Xylanibacter, Segatella, Hallela, Hoylesella* and *Leyella* (all previously *Prevotella*) species inhabiting oral cavity, urogenital tract, gut and rumen [11]. Their predicted targets corresponded closely to the available polysaccharide substrates in these habitats [11]. PULs were then studied in more detail in rumen species [12]. This study revealed their generalist/specialist strategies, diversity of PULs targeting the same broad substrate class (e.g. xylan) in different lineages and good agreement between predicted PULs and growth on various substrates [12]. It was however felt that the latter was rather indirect and clearer evidence confirming the PUL targets was needed. *Segatella bryantii* xylan PUL, whose components were already characterized in early studies [13] has already been confirmed using transcriptomic approach [14] and studied also in *S. copri* [15]. Recently also the arabinan, pectic galactan and inulin targeting PULs were confirmed in *S. copri* using transcriptomics and a genetic inactivation system specifically developed for this species [16]. We also recently obtained a naturally occurring rumen *Prevotella communis* mutant strain unable to use inulin, as opposed to other closely related strains, with deletions in homologous inulin PUL, which further confirms its target [17]. Here, we firstly present the transcriptomic identification of further hemicellulose and pectin targeting PULs using two species from *Xylanibacter* and *Segatella* genera to cover some of the PUL diversity. Since the chosen strains are generalists, we identify substrate preferences and evaluate whether the specific growth rate and extent of growth are its main driving force. Thirdly, we describe the use of β-glucan and xyloglucan in these strains in the absence of bioinformatically and transcriptomically identifiable PUL systems.

## Results

### Confirmation of predicted PULs using transcriptomics in *S. bryantii* TF1-3 and *X. ruminicola* KHP1

**Fig. 1:**
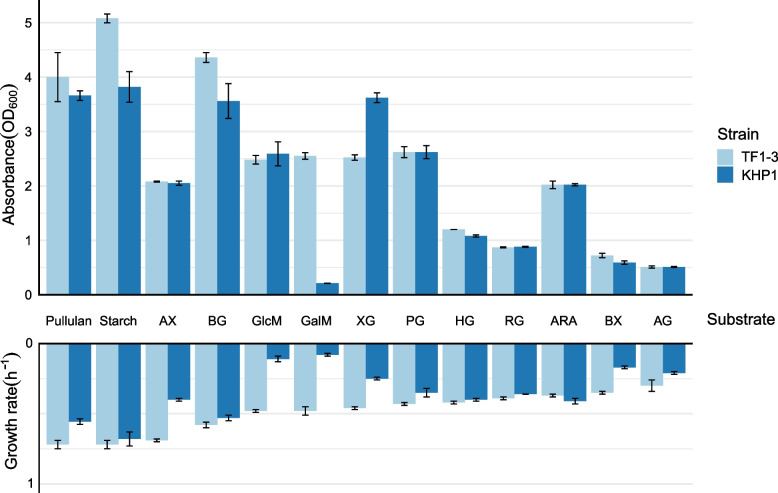
Maximum OD_600_ and specific growth rate values of *S. bryantii* TF1-3 and *X. ruminicola* KHP1 grown in different hemicelluloses, energy storage polysaccharides and pectin substrates. Upper part of the figure represents maximal density of *S. bryantii* TF1-3 (light blue) and *X. ruminicola* KHP1 (dark blue) cultures, respectively. Specific growth rates are represented on the bottom part with light blue and dark blue bars for *S. bryantii* TF1-3 and *X. ruminicola* KHP1, respectively. Data are averages and standard errors of four replicates. Polysaccharides used are indicated in the middle. The growth medium contained 0.5 % of either pullulan, starch, arabinoxylan (AX), β-glucan (BG), homogalacturonan (HG), pectic galactan (PG), glucomannan (Glum), galactomannan (Galm), xyloglucan (XG), rhamnogalacturonan (RG), arabinan, beechwood xylan (BX) or arabinogalactan (AG)

Our study focused on *S. bryantii* TF1-3 and *X. ruminicola* KHP1 as representative models. While previous studies predicted PULs in *X. ruminicola* KHP1 strain, the genome of *S. bryantii* TF1-3 was not examined [12]. We firstly investigated the presence of PULs previously found in other *S. bryantii* strains within the *S. bryantii* TF1-3 genome. Our analysis confirmed high similarity in PUL complement across all *S. bryantii* strains as observed earlier [12]. Table 1 provides an overview of the predicted PULs in both studied strains. Both strains showed large complements of predicted PULs targeting all major energy storage, hemicellulose and pectin polysaccharides found in the rumen or mammalian hindgut. For some substrates there were even two or more predicted PULs. They grew with most predicted substrates successfully ([12], Fig. 1). However, growth was also present in both strains with β-glucan (BG) and xyloglucan (XG), even though the PUL for β-glucan degradation was not predicted in *S. bryantii* TF1-3 and xyloglucan PUL was missing in *X. ruminicola* KHP1 (Table 1). Many other rumen *Xylanibacter* strains also lacked XG PUL but grew successfully on this substrate which was the only major false negative prediction of previous study [12].

**Table 1:** Overview of PULs and their associated CAZymes in *X. ruminicola* KHP1 and *S. bryantii* TF1-3. PULs relevant to this study are presented. For a full list, please see Tables S3-5 in [12]. Numbers indicate how many PULs were predicted for each substrate. Beside are the predicted CAZymes of these PULs. The information of genome size and number of CAZymes is also presented. Substrates: galacto/glucomannan (GalM/GlcM), arabinogalactan (AG), homogalacturonan (HG), rhamnogalacturonan (RG)

	Genome size	CAZymes (per Mb)	GalM/GlcM		β-glucan		Xyloglucan		AG		Arabinan		Starch		HG		RG		Xylan	
***X. ruminicola* KHP1**	3.41	198 (58.1)	0		3	GH3, GH5, GH16, GH36, CBM4, CBM48, CE1	0		1	GH43, GH105, PL12	2	GH2, GH30, GH35, GH43, CBM32	1	GH13, GH53, GH77, GH97, CBM20, CBM26	2	GH28, GH105, GH106, CE8, CE10, PL1	1	GH2, GH28, GH105, GH116, CE3, CE8, CE10, CE12, PL1, PL11	3	GH10, GH43, GH115, CBM4, CBM13, CBM6, CE1, CE6
***S. bryantii* TF1-3**	3.39	173 (51)	1	GH5, GH26, GH76, GH130, CE7	0		1	GH2, GH5, GH31	1		2		1		2		5		3

Here, we examined the utilization of 13 distinct substrates by the above two strains. Firstly, we reassessed the growth extent and specific growth rates using these substrates. Notably, we could not use the minimal medium designed for *S. bryantii* [18] in experiments since *X. ruminicola* KHP1 showed no growth in it. We opted instead for a modified DSMZ medium 330 used earlier [12] (please see methods) that in the absence of sugar substrate does not support any growth of ruminal *Xylanibacter* or *Segatella* strains. The growth characteristics for examined strains are in the Fig. 1. Based on data presented in Fig. 1 and defined cut-offs in the methods section, both bacteria excelled in growth on energy storage polysaccharides and β-glucan. *S. bryantii* TF1-3 grew well on several hemicelluloses (arabinoxylan, glucomannan, galactomannan and xyloglucan), while *X. ruminicola* KHP1 displayed lower growth rates on these substrates, but reached similar population densities, with exception of galactomannan. The growth of *X. ruminicola* KHP1 on this particular substrate was the worst of all the studied polysaccharides. On three out of five pectin substrates (homogalacturonan, rhamnogalacturonan, arabinogalactan) and on beechwood xylan the growth of both bacteria was poor. There is an interesting difference in growth on arabinoxylan and beechwood xylan. Recently, two PULs for xylan degradation were characterized in *S. copri* [19]. One of them is probably responsible for arabinoxylan degradation and was also found in *S. bryantii* TF1-3 (Additional file 1A; RG252_13075-13140) and *X. ruminicola* KHP1 (Additional file 1B; SAMN04487826_1268-88). The other PUL codes for GH10 that hydrolyzes glucuronosylated xylan polymers to xylooligosaccharides, thus may be important for beechwood xylan degradation [19]. *SusCD*-like genes of this PUL are present in *S. bryantii* TF1-3 (Additional file 1A; RG252_12240-45) and *X. ruminicola* KHP1 (Additional file 1B; SAMN04487826_1586-7) strains, but there are no CAZymes colocalized. In the case of *S. bryantii* TF1-3 most CAZymes from this PUL are present in arabinoxylan PUL, while in *X. ruminicola* KHP1 two of them are found in different places in genome.

**Fig. 2:**
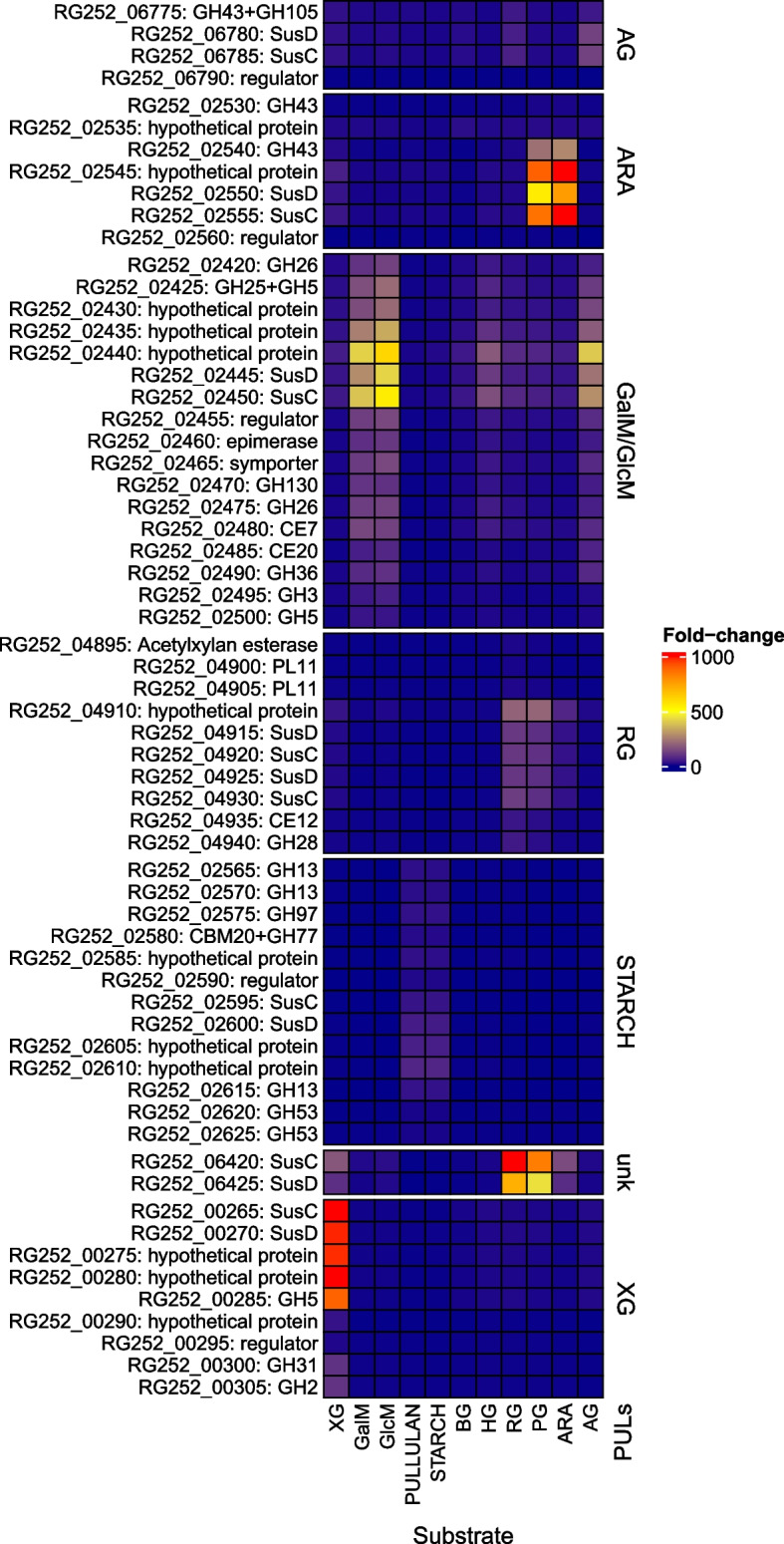
*S. bryantii* TF1-3 predicted PULs that were expressed in response to various polysaccharides. A heatmap showing PUL induction by hemicelluloses, energy storage polysaccharides and pectin substrates. Each rectangle represents fold-change (relative to glucose) of a gene in designated PUL (written at right side) and amounts to the values seen in the legend at the far-right side. Individual PULs are separated by white horizontal breaks. One PUL is named unk and represents a PUL that was not predicted by earlier bioinformatic analysis. On the left side are presented locus tags and products. Substrates used: xyloglucan (XG), galactomannan (GalM), glucomannan (GlcM), pullulan, starch, β -glucan (BG), homogalacturonan (HG), rhamnogalacturonan (RG), pectic galactan (PG), arabinan (ARA), arabinogalactan (AG)

**Fig. 3:**
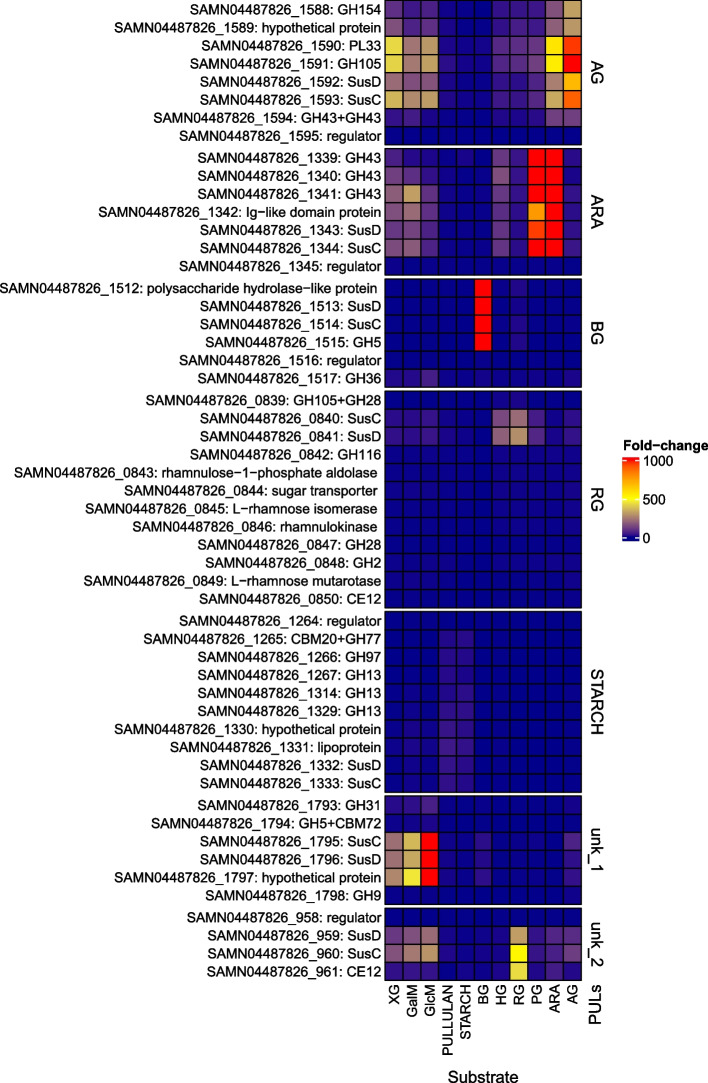
*X. ruminicola* KHP1 predicted PULs expressed in response to various polysaccharides. A heatmap showing PUL induction by hemicelluloses, energy storage polysaccharides and pectin substrates. Each rectangle represents fold-change (relative to glucose) of gene in designated PUL (written at right side) and amounts to the values seen in the legend at the far-right side. Individual PULs are separated by white horizontal breaks. Two PULs are named unk_1 and unk_2 respectively and represent PULs that were not predicted by earlier bioinformatic analysis. On the left side are presented locus tags and products. Substrates used are the same as in Fig. 2

Transcriptome analysis was then performed and a table specifying differentially expressed genes across distinct growth substrates was generated for both bacterial strains using the glucose dataset as reference. The complete log_2_-fold change (log_2_FC) values for genes surpassing a threshold of log_2_FC >3 and log_2_FC <-3 for at least one of the eleven substrates are presented in “Additional file 2” for *S. bryantii* TF1-3 and *X. ruminicola* KHP1. To facilitate a more effective visualization of the upregulated gene clusters, a heatmap was made with predicted PULs for each strain [12]. In Figs. 2 and 3 we present genes that exhibited the highest activation upon growth on their presumptive substrates for *S. bryantii* TF1-3 and *X. ruminicola* KHP1, respectively. The structures of these PULs are in ”Additional file 3”.

### Induction of predicted PULs in *S. bryantii* TF1-3

Some PULs (e.g. targeting starch and xyloglucan) were upregulated only by their targeted substrate, but others showed strong induction on several substrates (Fig. 2). This was the case of arabinan PUL that was strongly activated also by the pectic galactan. A similar phenomenon was also documented in the seminal PUL study in *Bacteroides* [10]. Moreover, these substrates also activated the rhamnogalacturonan PUL and previously unknown PUL (unk) which displayed highest activity on rhamnogalacturonan. Cross activation of rhamnogalacturonan PULs is in accordance with [10] and the fact that these substrates are both parts of rhamnogalacturonans. In the case of galacto/glucomannan PUL, where we also observed cross activation mainly by pectin substrates, explanation may not be straightforward. However this cross activation behavior is known in *Segatella* as the *S. copri* arabinoxylan PUL was activated efficiently also by other substrates [16]. The starch/pullulan PUL exhibited clear, but lower induction relative to other substrates implying that it was somewhat active even when growing bacteria on glucose in our system (Additional file 1A). Generally, the induction of *susCD*-like genes tended to surpass that of other PUL genes (Fig. 2). One of the predicted homogalacturonan PULs was induced to the greatest level when TF1-3 grew on homogalacturonan (Additional file 2A; RG252_04785-4810). However, the level of induction was modest and other PULs were activated to comparable level, thus its role in homogalacturonan degradation is inconclusive. On the other hand, the second predicted homogalacturonan PUL (Additional file 2A; RG252_05415-30) displayed low-level activity across most substrates further complicating drawing conclusions. Thus, in summary, seven PULs were confirmed using transcriptomics as most probable candidates for specific substrate degradation in *S. bryantii* TF1-3.

### Induction of predicted PULs in *X. ruminicola* KHP1

Illustrated in Fig. 3 is the induction of the predicted PULs in *X. ruminicola* KHP1. Starch PUL in *X. ruminicola* KHP1 is split by the 114,6 kb insertion (Additional file 3). Despite this, there is a moderate but exclusive induction of the whole PUL, quite similar to the one seen in *S. bryantii* TF1-3 upon growth on starch. The prediction of β-glucan PUL was also confirmed, as the PUL exhibited strong and exclusive activity when the bacterium was cultured on this particular substrate (Additional file 2B). We have found a pronounced activity of PUL, that was previously uncharacterized while growing *X. ruminicola* KHP1 on glucomannan and galactomannan substrates (Fig. 3, unk_1). Growth on these substrates activated most of the genes that were also active on xyloglucan containing growth medium. But the induction of this specific unknown PUL was so pronounced on glucomannan and galactomannan that its importance for growth on these substrates could be vital. Normally we would expect the presence of glycoside hydrolases from family 26 and 36 in galacto/glucomannan PUL, but it seems that this PUL is distinct to the one found in *Bacteroides* genus [20] and the one in *S. bryantii* TF1-3. Further examination revealed that the previously predicted rhamnogalacturonan PUL does indeed exhibit its highest activity when the bacterium is grown on rhamnogalacturonan, with somewhat lower levels of induction observed on other pectin substrates. Even higher activation of genes while growing *X. ruminicola* KHP1 on rhamnogalacturonan appeared in previously unknown PUL (Fig. 3, unk_2), which again displayed lower levels of induction on other substrates. Both, arabinan and pectic galactan strongly induced the predicted arabinan PUL, mirroring the behavior observed in *S. bryantii* TF1-3. Arabinan PUL was also activated at lower levels while growing on homogalacturonan and several other substrates. This was not observed in TF1-3 reflecting possible variations in activation behavior of these otherwise homologous PULs (Additional file 3). The predicted arabinogalactan PUL was surprisingly active on all substrates, except on starch/pullulan and β-glucan, yet showed the highest response on arabinogalactan substrate. Similar to what we observed in TF1-3, predicted homogalacturonan PUL was slightly induced on homogalacturonan substrate, while the other was induced at low levels by different substrates (Additional file 2B; SAMN04487826_00962-69 and SAMN04487826_01419-20 respectively). Since the induction of other PULs when bacteria were growing on homogalacturonan was higher, the importance of this latter PUL is questionable.

A significant number of the previously predicted PULs [12] have thus been upregulated when their presumptive targets were available. However, for some substrates, there were two or more PULs putatively involved in their degradation by the earlier study [12]. In the case of arabinan PUL, two were predicted in both strains, but just one was strongly induced in our experimental conditions (Additional file 4). A similar situation arose with predicted rhamnogalacturonan PULs. Some of them remained inert in their activity or exhibited low induction across various substrates. In addition to highly responsive β-glucan PUL in KHP1, two others were predicted but did not exhibit such activation when exposed to β-glucan substrate.

### Polysaccharide utilization in the absence of clear PULs.

There were two cases where both, bioinformatic analysis and transcriptomics failed to identify PULs involved in polysaccharide degradation and growth using its products. As mentioned above, *S. bryantii* TF1-3 achieved high growth rates and final population densities on β-glucan but no obvious PULs for its degradation could be predicted [12]. Thus, we expected to uncover a highly activated PUL for β-glucan utilization, which would differ drastically from the ones found in *Bacteroides.* Surprisingly, there was no such a PUL but some of other predicted PULs in *S. bryantii* TF1-3 were slightly activated, a background commonly seen in other substrates that strongly activated their respective PULs. Initially we reasoned that this was an artifact arising from yeast extract, used at 0.05 % in the growth medium as yeast cell wall contains some β-glucan. Thus, we conducted experiments utilizing minimal medium, which supports the growth of *S. bryantii* TF1-3, supplemented with glucose (reference medium) or β-glucan. Interestingly, the transcriptomic data remained consistent with the results obtained in the M330 medium indicating no clearly upregulated PUL (Additional file 5). Additionally, the β-glucan PUL was strongly induced in *X. ruminicola* KHP1 implying that yeast extract presence in the medium may not interfere drastically, at least for some PULs.

*X. ruminicola* KHP1 demonstrated a slow growth trajectory that resulted in remarkably high densities when grown on xyloglucan. Interestingly, the transcriptomic analysis revealed strong upregulation of numerous enzymes and *susCD*-like genes across an array of distinct PULs (arabinogalactan, unk_1, unk_2, arabinan and rhamnogalactan PUL) (Fig. 3, Additional file 2B). The notion that enzyme specificity and regulation of certain PULs may not be strictly oriented toward one substrate was already put forward [21]. In this case several PULs might bring about less rapid but thorough degradation of xyloglucan. Additionally, we explored the possibility of the T9SS system’s involvement in degradation of all studied substrates in both bacteria [7]. Recent research has shown the presence of such secretion systems in several *Bacteroides* species [7, 8] and their contribution to polysaccharide degradation. These systems are responsible for the secretion of various large proteins containing TIGR04183 domain. They are not part of PULs and have been found throughout the genome. Many of them encode different glycoside hydrolase and polysaccharide lyase domains and may represent an alternative mechanism for degradation of diverse polysaccharides, distinct from well-known PULs [7, 8]. A search for proteins encompassing the TIGR04183 domain was made (Additional file 6A). Among the genes that code for such proteins we found one (Additional file 1A, RG252_05960) that was upregulated on all polysaccharides and had the highest activity on β-glucan in *S. bryantii* TF1-3. In *X. ruminicola* KHP1 no genes coding proteins with TIGR04183 domain were activated by the polysaccharide substrates used. Consequently, it appears unlikely that the T9SS system is used for substrate degradation in *X. ruminicola* KHP1, but may be involved in degradation of β-glucan in *S. bryantii* TF1-3.

### Substrate preference

The varied growth characteristics of the two strains using different substrates (Fig. 1) gave us plenty of opportunities to explore their polysaccharide preferences. At first, we thought that preferred substrates would be the ones that enabled growth similar to growth that we predicted based on their monosaccharide content (Additional file 7, please see also methods section). We reasoned that if actual growth parameters were dissimilar this should indicate either lack of PULs or the difficulty of substrate degradation and import by the PUL products. The experiments that we conducted involved cultivation of each bacterium in a mixture of two substrates, present in equal concentrations. This firstly enabled a direct comparison of growth on mixed versus individual substrates. Subsequently, we collected RNA samples at several time points during the exponential growth phase and quantified gene expression of PUL marker genes, usually *susC*-like genes which were identified above. This approach aimed to determine whether maxOD and growth rate would influence the bacteria’s substrate preference as judged by gene activation.

**Fig. 4:**
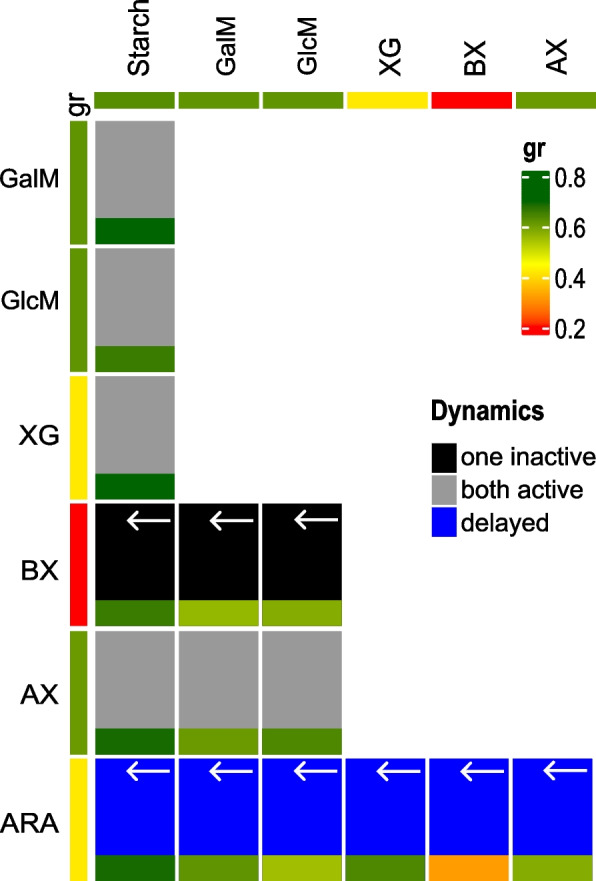
*S. bryantii* TF1-3 substrate preference. Composite heatmap with two values in each field showing PUL activation (dynamic) and growth rate in polysaccharide mixtures. At the side and at the top of the heatmap, the substrates and their respective PULs are indicated. Each heatmap field is separated in two rectangles. Smaller rectangles represent growth rates of bacteria in a mixture of two polysaccharides. Bigger rectangles represent dynamic of PUL induction in these mixtures. Arrows in dynamic rectangle point in the direction of PUL whose expression was delayed or PUL was inactive. Growth rate values are calibrated to the vertical bar (gr) in the legend, dynamic can be seen below it. Substrates used: starch, galactomannan (GalM), glucomannan (GlcM), xyloglucan (XG), beechwood xylan (BX), arabinoxylan (AX), arabinan (ARA)

**Fig. 5:**
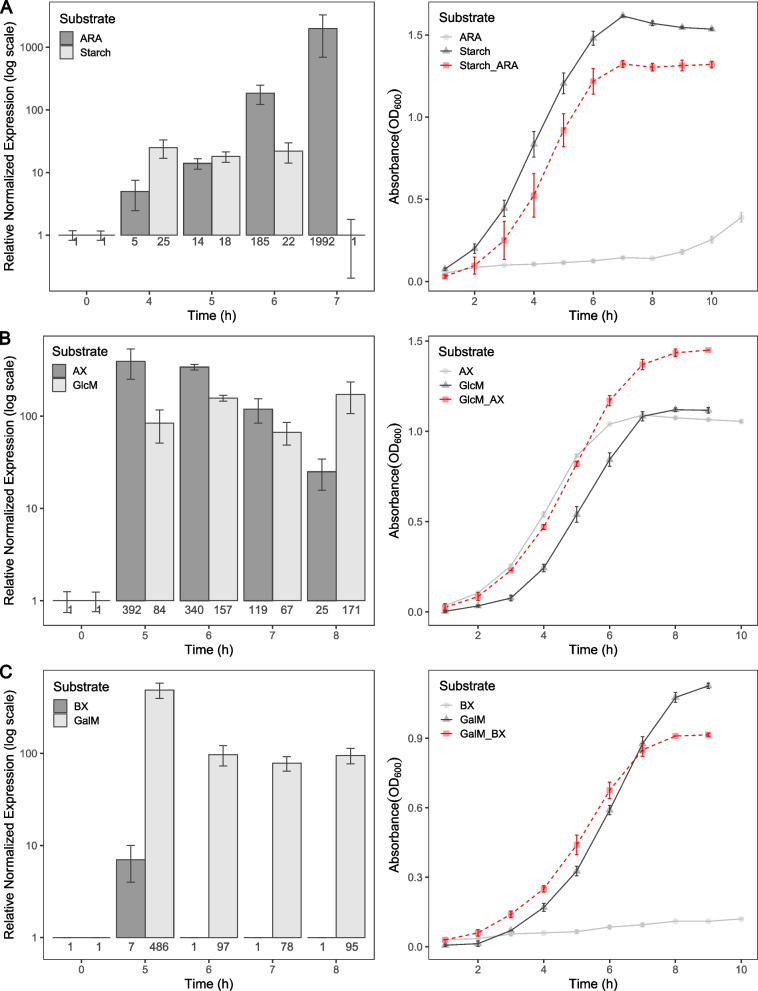
*S. bryantii* TF1-3 dynamics of PULs activation in mixtures of two polysaccharides. The left three graphs show relative normalized expression of *susC*-like genes of designated PULs over time to the quantity of transcripts on glucose (time 0) in the mixture of two polysaccharides present in equal concentrations. Values are written at the bottom of each bar. The expression of *susC*-like genes was measured with qRT-PCR. Right graphs show the growth curves in the mixture of two polysaccharides (0.3% total) and in each of the two (0.15%). Data are averages and standard errors of two biological replicates for growth curves and two technical repetitions with triplicates for RT-qPCR data. In (**A**) the example of “delayed” dynamic is presented. In (**B**) dynamic of “both active” and in (**C**) of “one inactive”. Substrates used: starch, galactomannan (GalM), glucomannan (GlcM), beechwood xylan (BX), arabinoxylan (AX), arabinan (ARA)

Surprisingly, the transcriptional activation of PULs in *S. bryantii* TF1-3 was consistently observed for both substrates in many cases, maintaining their activation throughout the observation period (Fig. 4). This was the case in most of the mixtures where starch was one of the two substrates. We also observed downregulation of the starch PUL at the final time point, possibly indicating rapid substrate degradation (Fig. 5A, Additional file 8A, B, I). Similarly, the simultaneous activation of both PULs was observed when the glucomannan or galactomannan were mixed with arabinoxylan indicating the importance of these hemicelluloses for bacterium (Fig. 5B, Additional file 8D). In the mixtures of arabinan with other substrates, *S. bryantii* TF1-3 arabinan PUL revealed a distinct pattern. We observed slower induction that reached its maximum only after several hours. At that time the activation of other PUL was sometimes already diminishing or was turned off completely, as in the case of starch PUL (Additional file 8E-G). The exception was the arabinan/xyloglucan mixture, where the induction was already high at the first time point (Additional file 8L). This could be due to arabinan PUL cross induction by xyloglucan, as can be seen in Fig. 2, resulting in a fold-change 4 times higher than on other substrates. Low level induction of arabinan PUL at first time-points may be explained by strong repression of arabinan PUL by glucose (Additional file 1A). This is a prerequisite to low levels of effector enzymes and SusCD-like binding proteins in PUL surveillance state and consequently possible slow signal amplification when grown on arabinan substrate. When TF1-3 was grown in mixtures containing beechwood xylan, the PUL for xylan utilization remained inactive or poorly expressed throughout the entire sampling period suggesting its low priority in *S. bryantii* TF1-3 (Fig. 5C, Additional file 8H, J). But when beechwood xylan was mixed with arabinan substrate, both PULs were active (Additional file 8K). This happened reproducibly several hours later than for other substrates tested, which is in line with previous results for arabinan. Interestingly xylan PUL did not remain inactive when *S. bryantii* TF1-3 was growing on mixtures with arabinoxylan, showing the ability to discriminate between these two substrates and indicating the preference for the arabinoxylan. In summary *S. bryantii* TF1-3 has several preferred substrates (starch, galacto/glucomannan, arabinoxylan and xyloglucan) that may be degraded simultaneously and whose PULs dominate in their activation relative the arabinan and beechwood xylan (please see methods section).

**Fig. 6:**
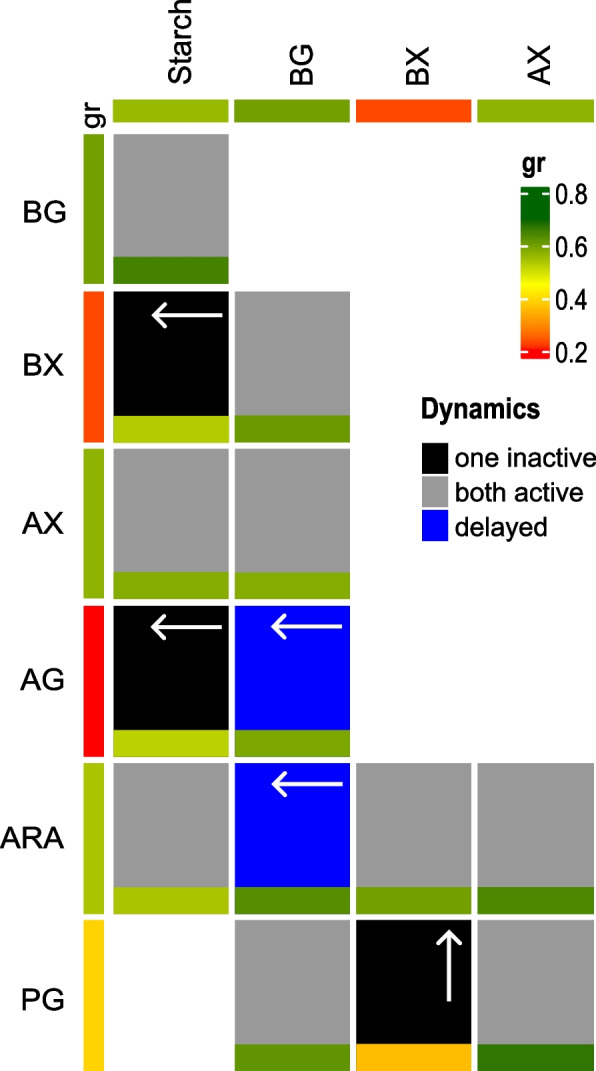
*X. ruminicola* KHP1 substrate preference. Composite heatmap with two values in each field showing PUL activation (dynamic) and growth rate in polysaccharide mixtures. At the side and at the top of the heatmap, the substrates and their respective PULs are indicated. Each heatmap field is separated in two rectangles. Smaller rectangles represent growth rates of bacteria in a mixture of two polysaccharides. Bigger rectangles represent dynamic of PUL induction in these mixtures. Arrows in dynamic rectangle point in the direction of PUL whose expression was delayed or PUL was inactive. Growth rate values are calibrated to the vertical bar (gr) in the legend, dynamic can be seen below it. Substrates used: starch, β -glucan (BG), beechwood xylan (BX), arabinoxylan (AX), arabinogalactan (AG), arabinan (ARA), pectic galactan (PG)

Similarly to *S. bryantii* TF1-3, *X. ruminicola* KHP1 growth on mixtures with starch often resulted in activation of both PULs (Fig. 6), but downregulation of starch PUL at final time-points was not observed (Additional file 9B-D, L, M). Both PULs were also active in mixtures of β-glucan with xylans and pectic galactan (Additional file 9H, I, K) and in the mixture of pectic galactan with arabinoxylan (Additional file 9G). Slow activation of arabinan PUL as seen in *S. bryantii* TF1-3 was observed only in the mixture of arabinan and β-glucan for *X. ruminicola* KHP1 (Additional file 9N). In mixtures of arabinan with xylans, both PULs were active (Fig. 6). Besides xylan PUL, which was not active in some mixtures with beechwood xylan like in *S. bryantii* TF1-3 (Additional file 9A, L), also the PUL for arabinogalactan did not activate or displayed delayed activation in *X. ruminicola* KHP1 despite the presence of this substrate in the mixtures (Additional file 9J, M). Thus, the preference list of *X. ruminicola* KHP1 that applies to our experimental conditions (mixture of two polysaccharides) seems to be β-glucan, starch, arabinoxylan, pectic galactan>arabinan>beechwood xylan>arabinogalactan. For both bacteria the preferred substrates were perhaps unsurprisingly the ones on which bacteria displayed good growth and were also in accordance with expected growth that was predicted from monosaccharide usage with the proposition that the depolymerization was not the limiting factor. The less preferred substrates (beechwood xylan, arabinogalactan) displayed poorer growth as we predicted.

## Discussion

Using transcriptomic approach in *S. bryantii* TF1-3 and *X. ruminicola* KHP1, we have confirmed a diverse array of PULs within their genomes and provided a catalogue of PULs supported both by bioinformatic and transcriptomic evidence. Among the seven predicted PULs in each of the studied bacteria, four of them (arabinan, arabinogalactan, starch/pullulan, unk - RG) exhibited homology of ≥60% in the SusCD-like protein sequences. Considering the gene organization, only the arabinan PUL is conserved in these species (Additional file 3). These differences strongly suggest distinct evolutionary pathways of PULs targeting specific substrates in these two bacterial species. For some substrates examined, e.g. homogalacturonan, the predicted PULs in both studied species were induced only moderately (but specifically) and drawing conclusions regarding the essentiality of these PULs for decomposition and growth has proven challenging. To gain a more certain understanding of such PUL targets, the implementation of genetic tools, as exemplified in the case of gut species *S. copri* [16], becomes essential. While such tools have been developed for *S. copri*, there are none at present for gene inactivation in any other ruminal *Segatella* and *Xylanibacter* species. The barriers toward gene introduction are high as reported in failed transformation attempts of *Prevotella* intermedia [22]. In this species several restriction systems were found to be active at the same time and high diversity of these systems was discovered in different strains. Furthermore, genetic tools would enable the study of the usage of substrates requiring the cooperative action of multiple PULs for degradation (pectic galactan, rhamnogalacturonan) [23] or for which no clear PUL bioinformatic prediction or transcriptomic signal is obtained, e.g. xyloglucan usage of *X. ruminicola* KHP1.

Overall, based on fold-change relative to growth on glucose (Additional file 2) we observed strong induction of *susCD*-like genes within most PULs, while the activation of effector genes was generally less prominent. This seems to not be the case in *Bacteroides* species [10, 24–26]. Considering all this, the transcriptomic search for highly induced proteins with TIGR04183 domain that could potentially be secreted by the T9SS system is limited. Thus, the possibility for utilization of T9SS system for substrate degradation in this genus remains a subject of debate.

The absence of gene induction of several predicted PULs (Additional file 4) may be attributed to the specificity of PUL activation. It is plausible that the specific glycosidic linkages between monosaccharides within a polysaccharide molecule or various substitutions (methylations, acetylations, sulfation) play a vital role in dictating the activation of particular PULs [10]. This hypothesis suggests that even a slight variance in the molecular structure of substrate might be a critical determinant. To further expand our understanding of the presence and activation dynamics of PULs, a broader array of substrates, and potentially those sourced from natural environments is needed.

Research made in *Bacteroides* proposes that these bacteria possess the ability to prioritize the utilization of polysaccharides [27–29]. This extends also to competitive environment where the preferences of the bacteria for polysaccharides remain the same even if it leads to competition for the same substrate which they both prefer [30]. In some cases the repression of use of several polysaccharides (arabinan, arabinogalactan) by glucose was observed [29] and involvement of sRNAs in substrate prioritization was proposed [31]. While growing *S. bryantii* TF1-3 and *X. ruminicola* KHP1 in mixtures of two hemicelluloses or energy storage polysaccharide both PULs were active in more than half of the cases. The simultaneous degradation could allow these bacteria to obtain substrates that are cross-linked and release them from the cell wall. Alternatively, the primary degradation of plant material can be performed by other bacteria and *X. ruminicola* and *S. bryantii* activate different PULs to degrade as much as possible of soluble polysaccharides that are released to the environment. Despite this, some results indicate clear preference for specific substrates over others. This was the case when bacteria were grown in the mixtures containing one pectin substrate or in the mixtures with beechwood xylan. Particularly while growing bacteria in the mixtures with arabinan or arabinogalactan we observed mild repression of these two PULs by several substrates as was observed before in *Bacteroides* [29].

Counterintuitively, we observed cases where growth in mixtures resulted in poorer growth compared to the growth observed when a single polysaccharide present in the mixture was used as the sole substrate at same concentration. A notable example is the growth of *S. bryantii* TF1-3 on galacto/glucomannan in a mixture with arabinogalactan substrate on which both bacteria displayed poor growth (Fig. 1). The growth curve in the mixture indicated a distinct lack of glucomannan use, as evidenced by its significant deviation from the growth pattern observed when glucomannan was the sole substrate (Additional file 10A, B). Conversely, the presence of arabinogalactan in the mixture did not repress the utilization of galactomannan despite that its PUL is the same. Similar behavior was observed while growing *X. ruminicola* KHP1 in same mixtures. The growth in mixtures was worse than on sole substrates (Additional file 10C, D).

## Conclusion

Results of our transcriptomic analysis provide findings upon which future experiments can be built, especially those focused on molecular mechanisms underlying regulation of polysaccharide utilization. Our study also serves as foundational step for investigations of *Xylanibacter* and *Segatella* behavior when grown together in mixtures of more polysaccharides, which would show us polysaccharide utilization strategies in even more complex environments. It seems that *Xylanibacter* and *Segatella* are more generalists in nature than *Bacteroides* and can degrade more polysaccharides at the same time which could explain their persistence in gut environment for a long time.

## Methods

### Bacterial strains and growth on pure glycans

Both used strains were obtained from cow rumen: *X. ruminicola* KHP1 was isolated in New Zealand during the Hungate 1000 project [32] while the *S. bryantii* TF1-3 in Sweden in 1990 [4]. The type strains of these species have been isolated several decades ago and it was felt that strains with considerably less passages would better reflect the original species nature. The strains were routinely cultivated in Hungate tubes at 37°C in the rumen fluid reinforced medium M2 [33] until reaching the stationary phase. For growth tests and isolation of RNA, they were sub-cultured into liquid modified DSMZ medium 330 [34] containing glucose or one of various hemicellulose, pectin or energy storage polysaccharide substrates at final concentration of 0.5% for growth tests on pure glycans for RNAseq analysis and 0.15% for growth test needed for analysis of substrate preferences for qRT-PCR (Additional file 6B). The *S. bryantii* TF1-3 was also grown in anaerobic defined minimal medium for *Segatella* with either glucose or β-glucan as the sole carbohydrate source [18]. Spectrophotometric measurements at 600 nm were taken at 1-hour intervals to monitor growth. In order to lengthen the linear range of growth curves for calculation of growth rates, dilutions of cultures were made and immediately measured when the OD values reached values ≥0.8. The growth rate and maximum optical density (maxOD) values for each strain on each substrate were calculated from the acquired growth curves (Additional file 6C). maxOD refers to highest OD values measured, while growth rate values were derived using the formula [*(ln(A*_*max*_*)-ln(A*_*min*_*))/T*_*max*_*-T*_*min*_] with minimum and maximum optical density values in exponential phase. We had 4 biological replicates. Growth characteristics of bacterial growth were then divided in three categories (poor, good and excellent) based on both growth rates and population densities. Growth on substrate was deemed excellent if the growth rate was ≥0.5/h and final population density was ≥3 OD units. If the growth rate was <0.5/h and population density ≤1.5 OD units the growth was poor. In other cases, the growth was defined as good. We also constructed weights for calculation of expected growth rate and population density using polysaccharide substrates based on the composition of each polysaccharide provided by the manufacturer (Additional file 6D). We than multiplied each weight with growth rate or population density on each monosaccharide and summed them.

### Isolation of DNA and genome assembly of *S. bryantii* TF1-3

Whole genomic DNA isolation of *S. bryantii* TF1-3 was done with Monarch Genomic DNA Purification Kit (New England biolabs) and the quantity of the extracted DNA was assessed using the Spark 10M multifunctional microplate reader using the QuantiFluor dsDNA System (Promega). Whole genome sequencing of *S. bryantii * TF1-3 strain was performed by Novogene with Illumina NovaSeq 6000 (2x150bp, 10M). To ensure data quality, reads underwent quality filtering and adapter removal via Trimmomatic v0.39 [35]. The genome assembly was done with SPAdes genome assembler v3.15.3 [36] and deposited in GenBank under the Bioproject PRJNA800605 and accession number JAVIXB000000000. Genome annotation for local analysis was executed using prokka 1.14.6 [37].

### Isolation of RNA for RNA-seq analysis

Strains were grown in liquid minimal or modified DSMZ medium 330 containing different substrates as explained before, until they reached OD value of 0.3-0.5. 1.5 mL of each triplicate was removed and immediately combined with 3 mL of RNAprotect bacteria reagent (Qiagen). RNA was purified using RNeasy Mini Kit (Qiagen) with the on-column DNase treatment. The quantity of the isolated RNA was determined using Spark 10M multifunctional microplate reader with Quant-iT^TM^ RNA Assay Kit (Thermo Scientific).

### Bioinformatics/RNA-seq analysis/statistical analysis

RNA of each triplicate culture grown on different substrates was sent for library preparation and sequencing, either to Microsynth or Novogene (Additional file 6E). Approximately 10 to 20 M single end reads or 10 M paired end reads per sample were obtained. Trimmomatic v0.39 [35] was used to perform quality filtering and adapter removal of the reads. The genomes of both bacteria were indexed, and the reads were aligned upon them using the BWA tool 0.7.17-r1188 [38]. We used genome of *X. ruminicola* KHP1 that is available in GenBank under Bioproject PRJEB15822 and accession number FNJZ01000001-6. The generated files were sorted using samtools v 1.11 [39], and gene counts were determined from the aligned data with featureCounts v 2.0.0 [40]. Differential gene expression analysis for each strain separately was performed with R 4.1.2, using edgeR [41] and limma packages [42]. To generate Additional file 1 the counts were normalized to transcripts per million values (TPM), while for sample normalization to calculate log_2_FC values EdgeR’s TMM method was utilized. Reads were then filtered with FiltrbyExpr in edgeR. A simple generalized linear model was generated using filtered data from all samples, and contrasts based on carbon source were used to identify differentially expressed genes.

### Transcriptional analysis of PUL genes by qPCR

Strains were grown in Hungate tubes at 37°C in the rumen fluid reinforced medium M2 until reaching the stationary phase and then sub-cultured into liquid modified DSMZ medium 330 containing mixture of two different polysaccharides at final concentration of 0.3% (each 0.15%). Cells were harvested at different time points in exponential phase, treated with RNAprotect (Qiagen), and stored at -20°C for further processing. Total RNA was extracted from cells using Total RNA Miniprep Kit (Monarch) and samples were also treated with DNase I (Thermo Scientific). The reverse transcription was performed using RevertAid Reverse Transcriptase (Thermo Scientific) according to the manufacturer’s instructions. cDNA quantification was performed with a Bio-Rad CFX96 Real-Time System using KAPA SYBR® Fast qPCR master mix (Kapa Biosystems, Inc., Wilmington, MA) for 40 cycles of 95°C for 3 s and 60°C for 30 s. Statistical analysis of qPCR data was done with Bio-Rad CFX Maestro™ Software. All transcript levels were normalized based on the abundance of 16S rRNA, with transcript levels of strains grown on glucose (OD = 0.6) serving as references. The qPCR essays were done in triplicates with two technical repetitions. The primers targeted the previously transcriptomically validated *susC* genes and are provided in “Additional file 6F”.

### Substrate preferences

The order of substrate preferences in each strain was obtained by awarding the points to each PUL on tested substrate combinations based on its dynamic. PUL was awarded 0 points if *susC-*like gene of a PUL was inactive. This was the case when induction was ≤5 on average through all the time points. PUL’s dynamic was delayed if the activation of *susC*-like gene was gradual and was thus awarded 0.5 points. PUL was awarded 1 point if its *susC*-like gene was active during all time points with exception of last time point in the case of starch where starch was presumably already depleted. The summed points of each PUL were then divided with number of assays that were made with the PUL substrate and compared.

### Supplementary Information

Supplementary Material 1.

Supplementary Material 2.

Supplementary Material 3.

Supplementary Material 4.

Supplementary Material 5.

Supplementary Material 6.

Supplementary Material 7.

Supplementary Material 8.

Supplementary Material 9.

Supplementary Material 10.

## Data Availability

The genomes analyzed during the current study are available in GenBank under the Bioproject PRJNA800605 and accession number JAVIXB000000000 for S. bryantii TF1-3. Raw and processed RNAseq data discussed in this publication have been deposited in NCBI’s Gene Expression Omnibus and are accessible through GEO Series accession number GSE262719 (https://www.ncbi.nlm.nih.gov/geo/query/acc.cgi?acc=GSE262719).

## Abbreviations

PUL: Polysaccharide utilization locus
CAZyme: Carbohydrate acting enzyme
qRT-PCR: Reverse transcription quantitative polymerase chain reaction
TPM: Transcripts per million
