# Supplementary material for: Transcriptomic analysis of polysaccharide utilization loci reveals substrate preferences in ruminal generalists Segatella bryantii TF1-3 and Xylanibacter ruminicola KHP1
Source: BMC Genomics. 2024 May 20;25:495. doi: 10.1186/s12864-024-10421-z (PMC11107044; doi:10.1186/s12864-024-10421-z)

Additional file 3: Schematic of *S. bryantii* TF1-3 and *X. ruminicola* KHP1 PULs involved in degradation of plant polysaccharides. All genes in PULs are colored as described in the color legend written on the right side and are present above the line (contig number on the left) which represents the place of PUL in genome of each bacteria. In the arrows are written families of glycoside hydrolases (GHs), polysaccharide lyases (PLs) and carbohydrate esterases (CEs). *susC*-like genes from all PULs are aligned. Presented PULs: arabinogalactan (AG), arabinan/pectic galactan (ARA), beta-glucan (BG), galacto/glucomannan (GalM/GlcM, unk_1), starch, rhamnogalacturonan (RG, unk, unk_2), xyloglucan (XG).

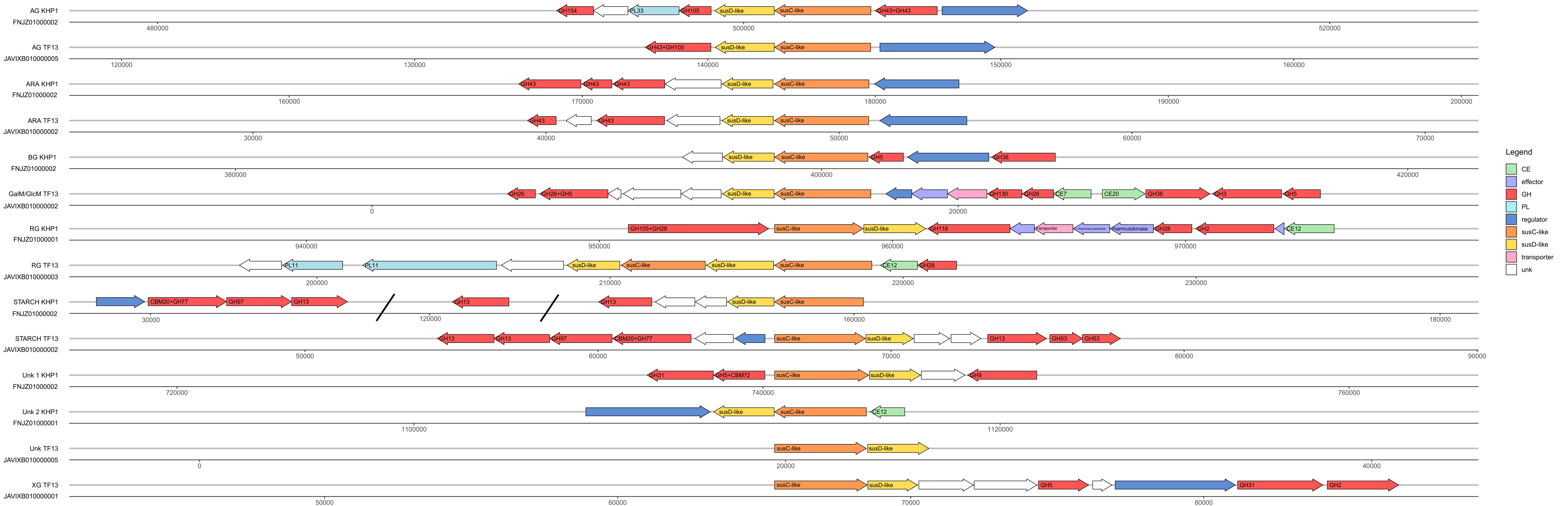

Supplement: Supplementary file 3 — Supplementary Material 3. [file 12864_2024_10421_MOESM3_ESM.pdf]
