# Supplementary material for: Transcriptomic analysis of polysaccharide utilization loci reveals substrate preferences in ruminal generalists Segatella bryantii TF1-3 and Xylanibacter ruminicola KHP1
Source: BMC Genomics. 2024 May 20;25:495. doi: 10.1186/s12864-024-10421-z (PMC11107044; doi:10.1186/s12864-024-10421-z)

Additional file 7: Maximum measured and predicted OD and growth rate values of *S. bryantii TF1-3* (blue) and *X. ruminicola* KHP1 (green) grown in different hemicelluloses, energy storage polysaccharides and pectin substrates. The upper part of graph represents final population densities of bacteria, graph below represents growth rate values. Expected values for both maximal density and growth rate are represented in darker shade of blue and green for *S. bryantii* TF1-3 and *X. ruminicola* KHP1, respectively. Absorbance was measured at 600 nm. Data are averages and standard error of four replicates. On the line between two graphs are indicated polysaccharides used. The growth medium contained 0.5 % of each substrate. Substrates used are the same as in Fig. 1.

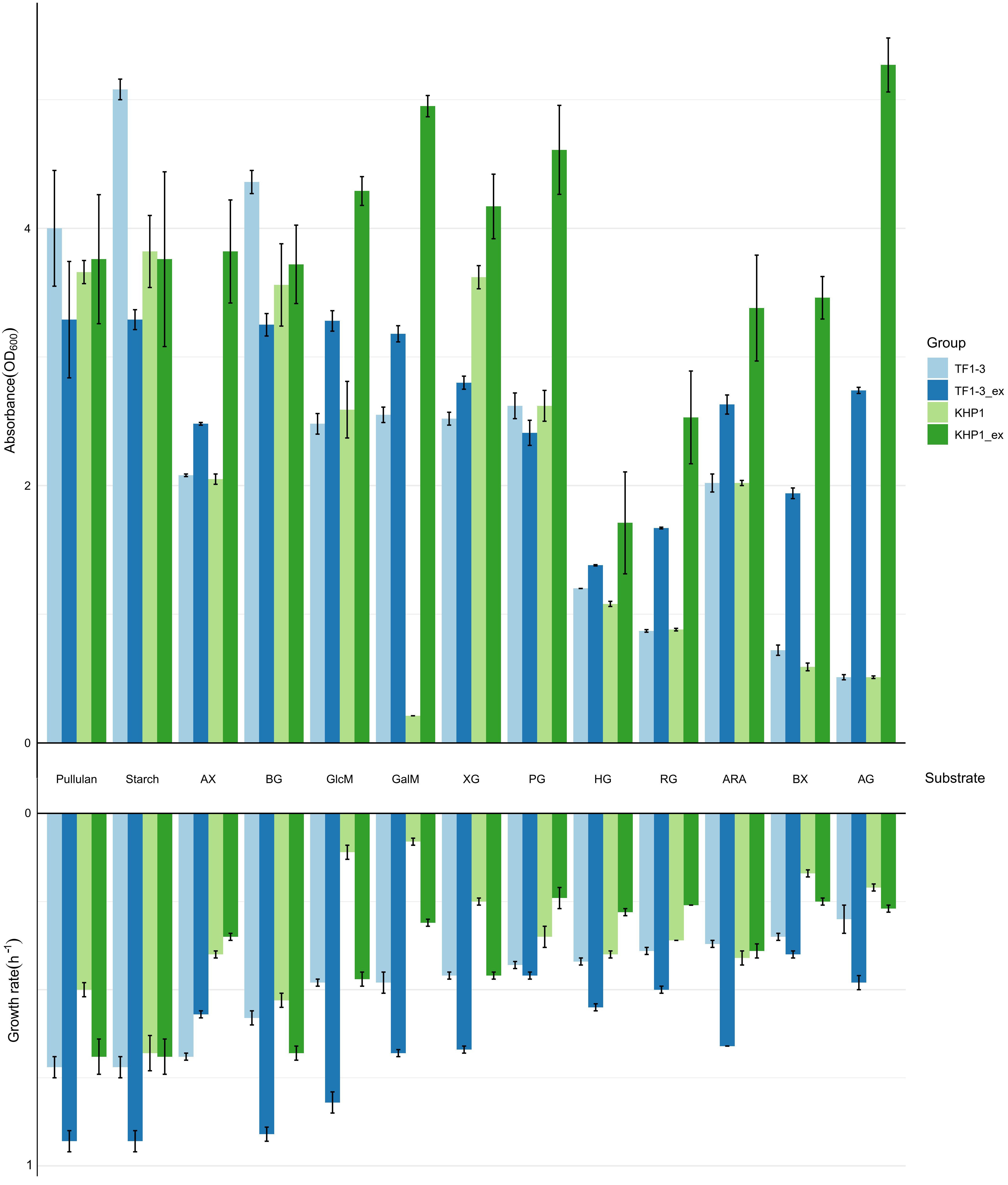

Supplement: Supplementary file 7 — Supplementary Material 7. [file 12864_2024_10421_MOESM7_ESM.pdf]
