# Supplementary material for: Transcriptomic analysis of polysaccharide utilization loci reveals substrate preferences in ruminal generalists Segatella bryantii TF1-3 and Xylanibacter ruminicola KHP1
Source: BMC Genomics. 2024 May 20;25:495. doi: 10.1186/s12864-024-10421-z (PMC11107044; doi:10.1186/s12864-024-10421-z)
Supplement: Supplementary file 8 — Supplementary Material 8. [file 12864_2024_10421_MOESM8_ESM.pdf]

Additional file 8: All reverse-transcription qPCR and growth tests of *S. bryantii* TF1-3 grown in mixtures of two polysaccharides that are presented in a heatmap (Fig. 4). Left graphs show relative normalized expression of *susC*-like genes of designated PULs over time relative to quantity of transcripts on glucose (time 0) in the mixture of two polysaccharides present in equal concentrations (0.15% each). Values are written at the bottom of each bar. The expression of *susC*-like genes was measured with qPCR. Right graphs show growth of *S. bryantii* TF1-3 in each of the two polysaccharides (0.15%) and in the mixture of both (0.3% - each 0.15%). Data are averages and standard errors of two biological replicates. Substrates used: starch, galactomannan (GalM), glucomannan (GlcM), xyloglucan (XG), beechwood xylan (BX), arabinoxylan (AX), arabinan (ARA).

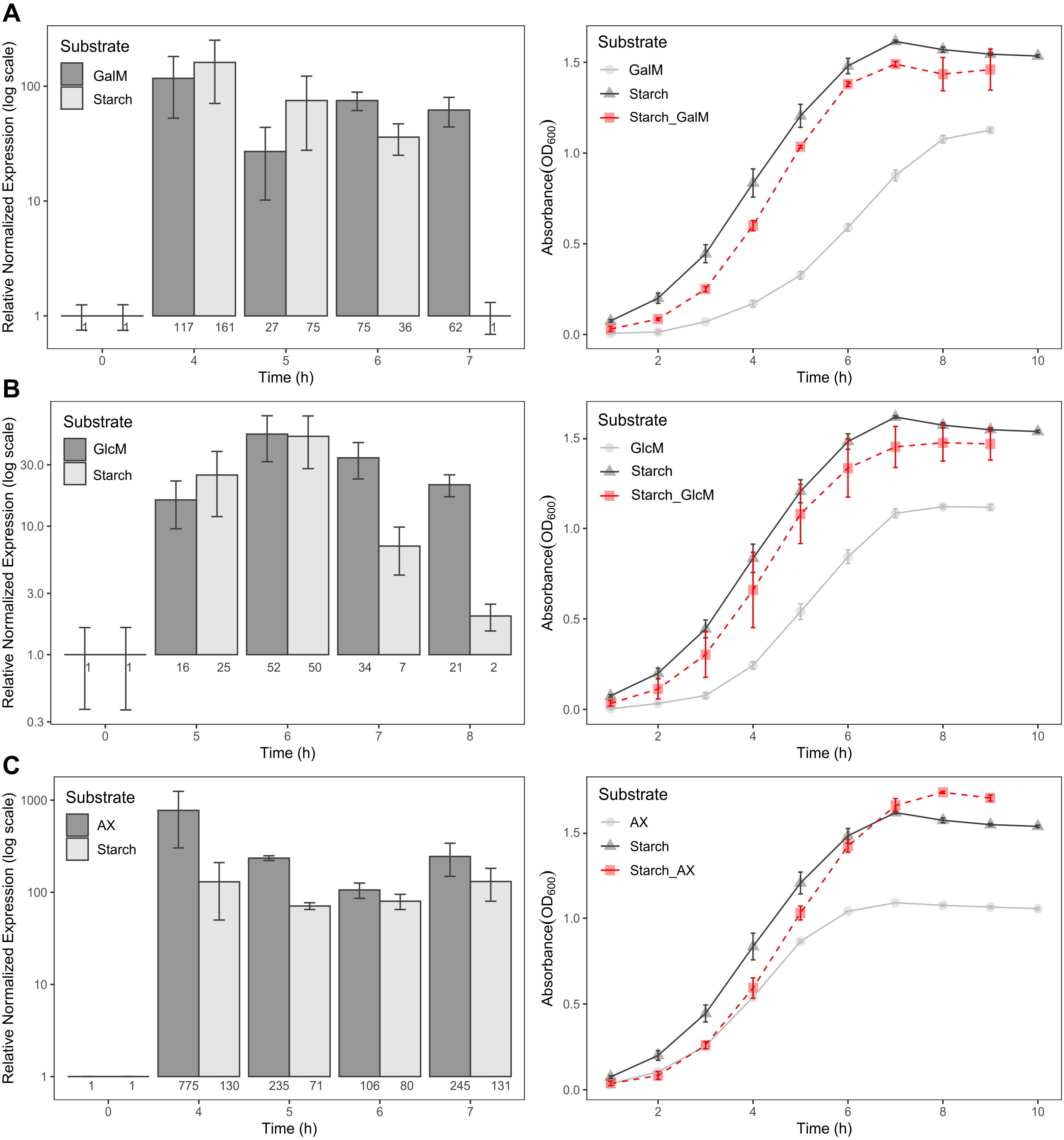

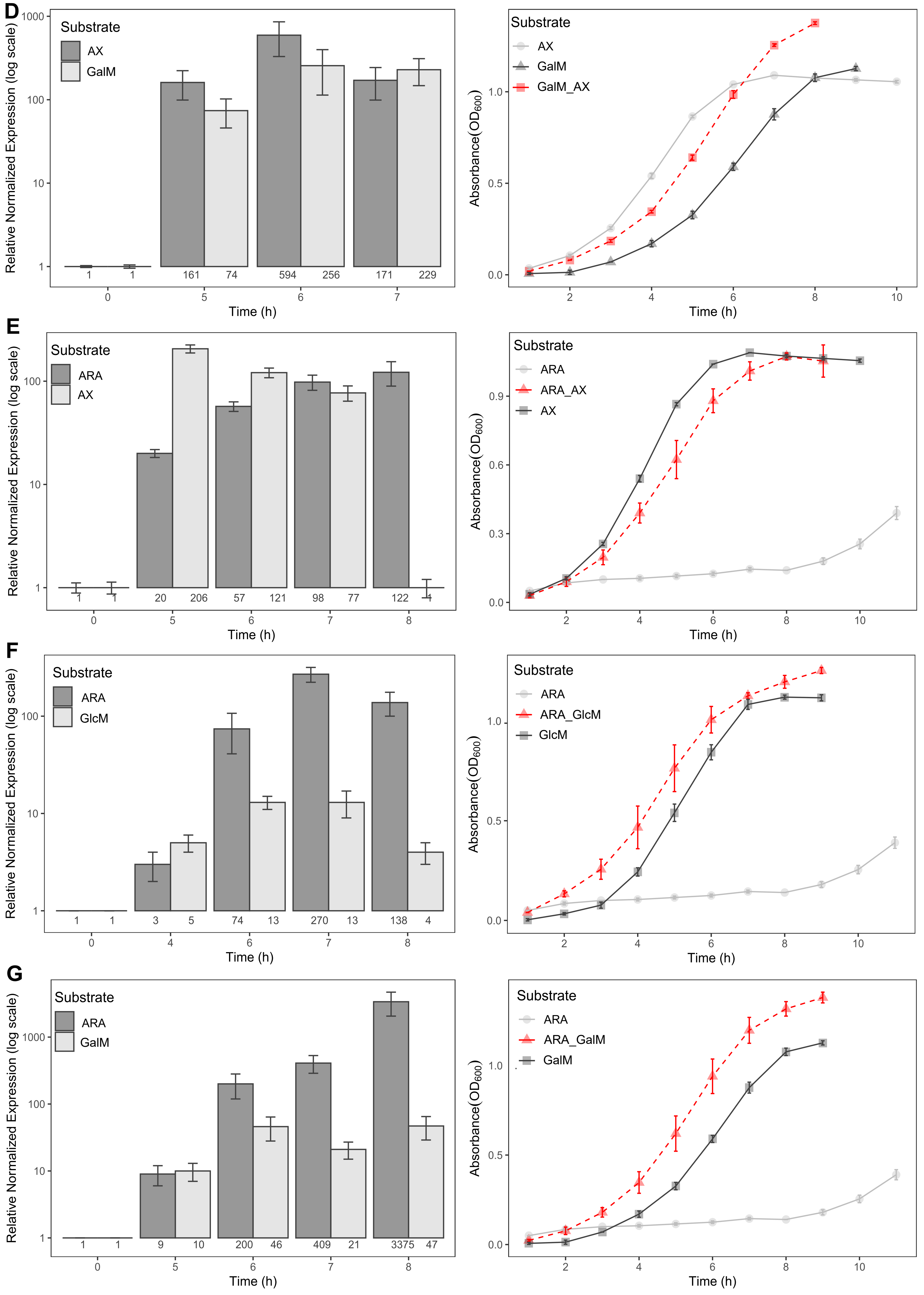
D
Substrate
AX
GalM
Relative Normalized Expression (log scale)
1000
100
10
1
1 1 161 74 594 256 171 229
0 5 6 7
Time (h)
Substrate
AX
GalM
GalM_AX
Absorbance(OD600)
1.0
0.5
0.0
2 4 6 8 10
Time (h)
E
Substrate
ARA
AX
Relative Normalized Expression (log scale)
100
10
1
1 1 20 206 57 121 98 77 122 1
0 5 6 7 8
Time (h)
Substrate
ARA
ARA_AX
AX
Absorbance(OD600)
0.9
0.6
0.3
0.0
2 4 6 8 10
Time (h)
F
Substrate
ARA
GlcM
Relative Normalized Expression (log scale)
100
10
1
1 1 3 5 74 13 270 13 138 4
0 4 6 7 8
Time (h)
Substrate
ARA
ARA_GlcM
GlcM
Absorbance(OD600)
1.0
0.5
0.0
2 4 6 8 10
Time (h)
G
Substrate
ARA
GalM
Relative Normalized Expression (log scale)
1000
100
10
1
1 1 9 10 200 46 409 21 3375 47
0 5 6 7 8
Time (h)
Substrate
ARA
ARA_GalM
GalM
Absorbance(OD600)
1.0
0.5
0.0
2 4 6 8 10
Time (h)

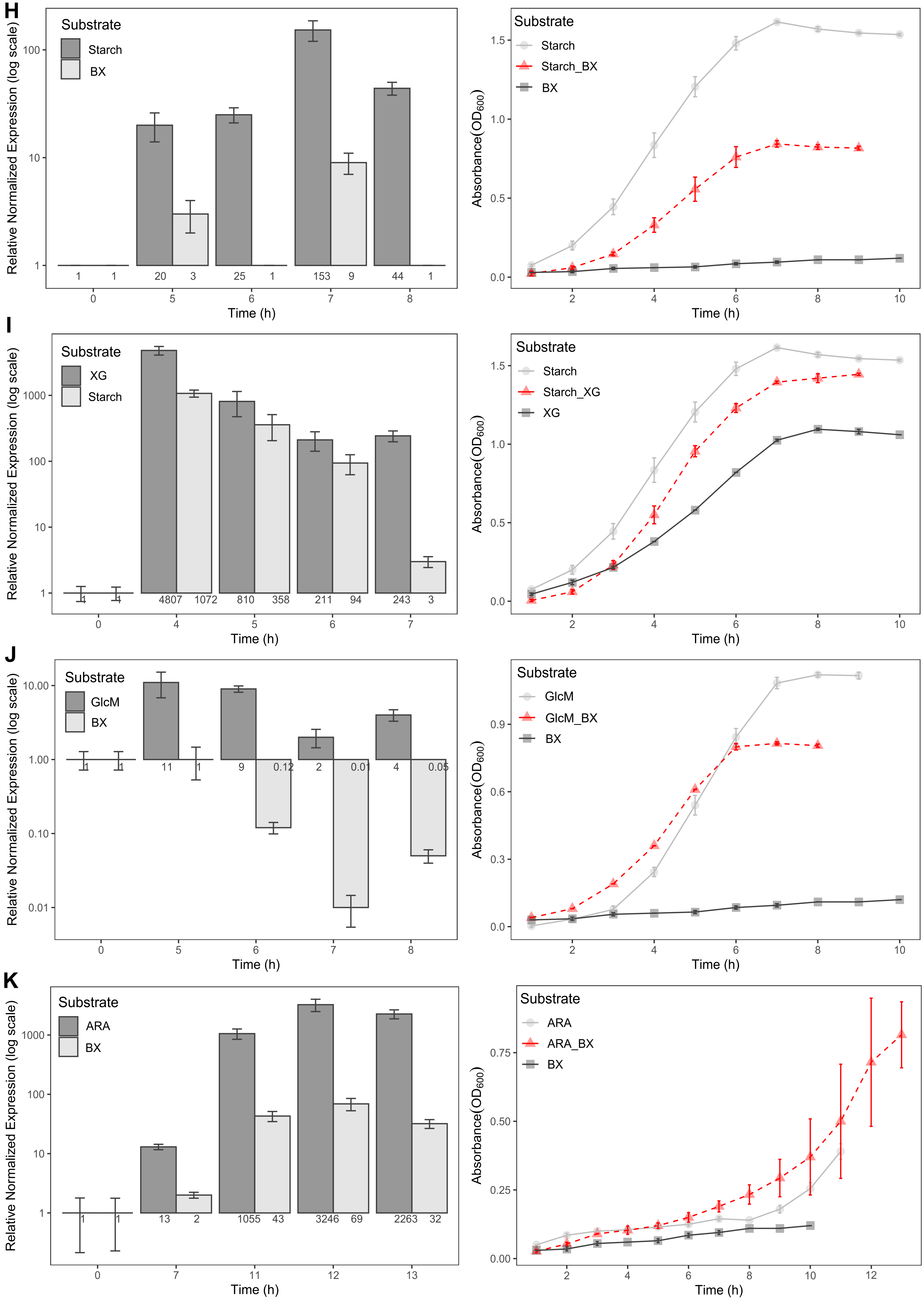

H
Substrate
Starch
BX
Relative Normalized Expression (log scale)
100
10
1
1 1 20 3 25 1 153 9 44 1
0 5 6 7 8
Time (h)
Substrate
Starch
Starch_BX
BX
Absorbance(OD600)
1.5
1.0
0.5
0.0
2 4 6 8 10
Time (h)
I
Substrate
XG
Starch
Relative Normalized Expression (log scale)
1000
100
10
1
1 1 4807 1072 810 358 211 94 243 3
0 4 5 6 7
Time (h)
Substrate
Starch
Starch_XG
XG
Absorbance(OD600)
1.5
1.0
0.5
0.0
2 4 6 8 10
Time (h)
J
Substrate
GlcM
BX
Relative Normalized Expression (log scale)
10.00
1.00
0.10
0.01
1 1 11 1 9 0.12 2 0.01 4 0.05
0 5 6 7 8
Time (h)
Substrate
GlcM
GlcM_BX
BX
Absorbance(OD600)
0.9
0.6
0.3
0.0
2 4 6 8 10
Time (h)
K
Substrate
ARA
BX
Relative Normalized Expression (log scale)
1000
100
10
1
1 1 13 2 1055 43 3246 69 2263 32
0 7 11 12 13
Time (h)
Substrate
ARA
ARA_BX
BX
Absorbance(OD600)
0.75
0.50
0.25
0.00
2 4 6 8 10 12
Time (h)

L

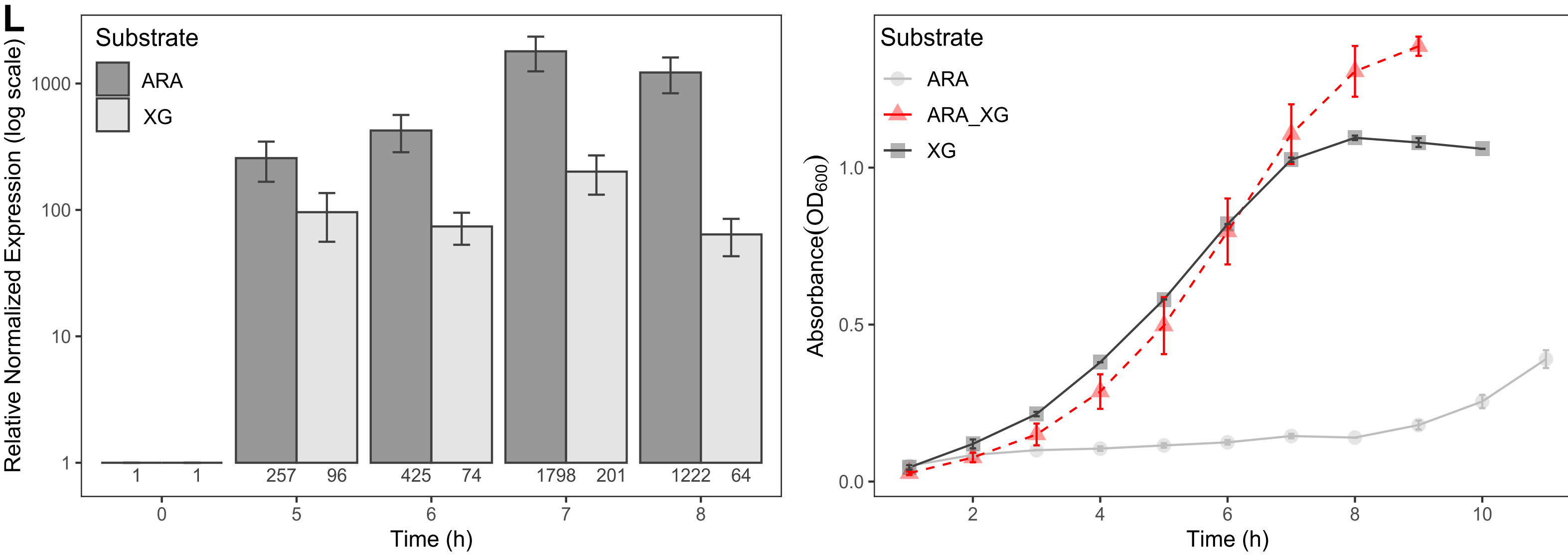
Substrate
ARA
XG
Relative Normalized Expression (log scale)
1000
100
10
1
1 1 257 96 425 74 1798 201 1222 64
0 5 6 7 8
Time (h)
Substrate
ARA
ARA_XG
XG
Absorbance(OD_{600})
1.0
0.5
0.0
2 4 6 8 10
Time (h)
