# Supplementary material for: Transcriptomic analysis of polysaccharide utilization loci reveals substrate preferences in ruminal generalists Segatella bryantii TF1-3 and Xylanibacter ruminicola KHP1
Source: BMC Genomics. 2024 May 20;25:495. doi: 10.1186/s12864-024-10421-z (PMC11107044; doi:10.1186/s12864-024-10421-z)
Supplement: Supplementary file 9 — Supplementary Material 9. [file 12864_2024_10421_MOESM9_ESM.pdf]

Additional file 9: All reverse-transcription qPCR and growth test of *X. ruminicola* KHP1 grown in mixture of two polysaccharides that are presented in a heatmap (Fig. 6). Left graphs show relative normalized expression of *susC*-like genes of designated PULs over time relative to quantity of transcripts on glucose (time 0) in the mixture of two polysaccharides presented in equal concentrations (0.15% each). Values are written at the bottom of each bar. The expression of *susC*-like genes was measured with qPCR. Right graphs show growth of *X. ruminicola* KHP1 in each of the two polysaccharides (0.15%) and in the mixture of both (each 0.15%). Data are averages and standard error of two biological replicates. Substrates used: starch, β-glucan (BG), beechwood xylan (BX), arabinoxylan (AX), arabinogalactan (AG), arabinan (ARA), pectic galactan (PG).

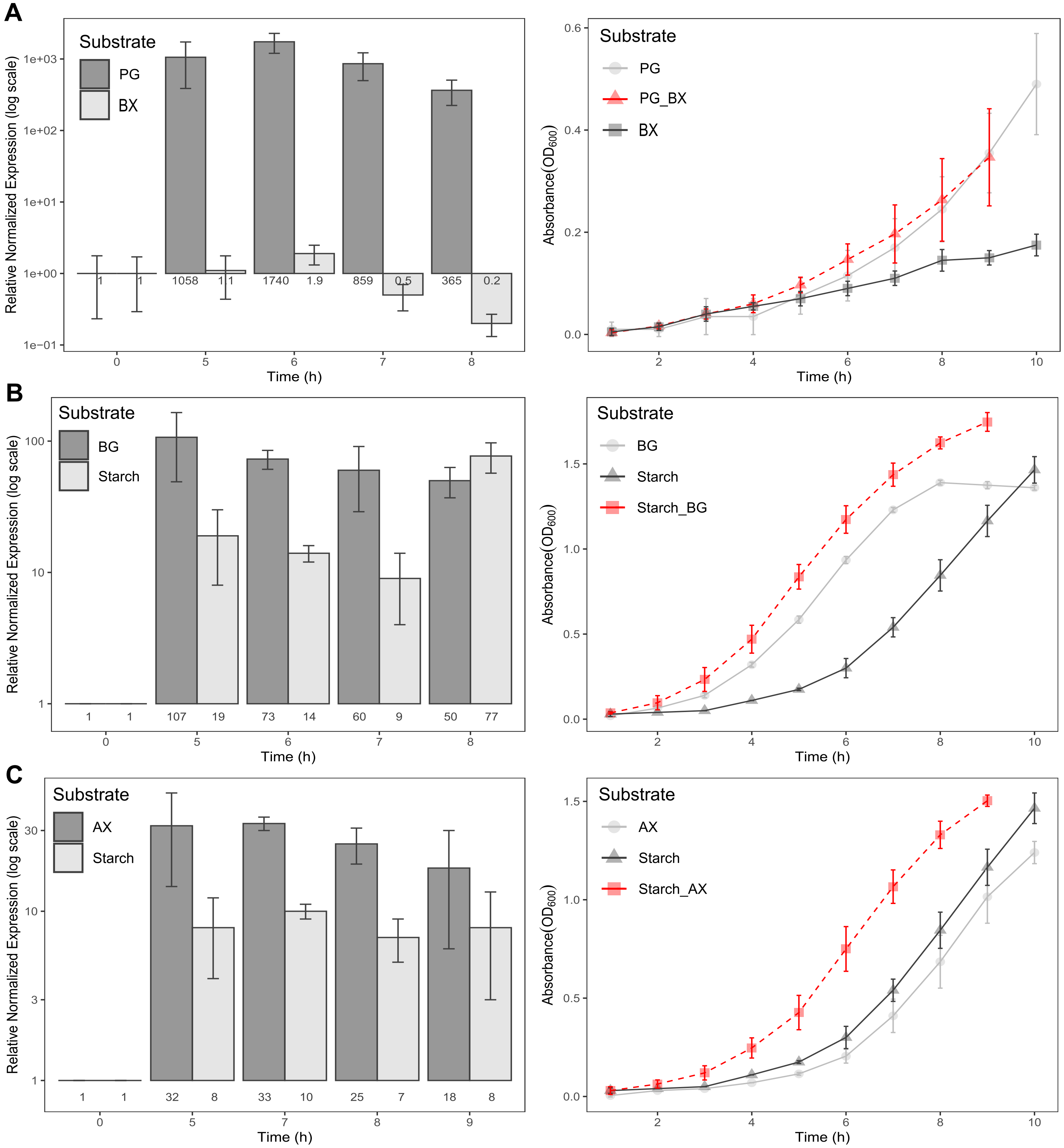

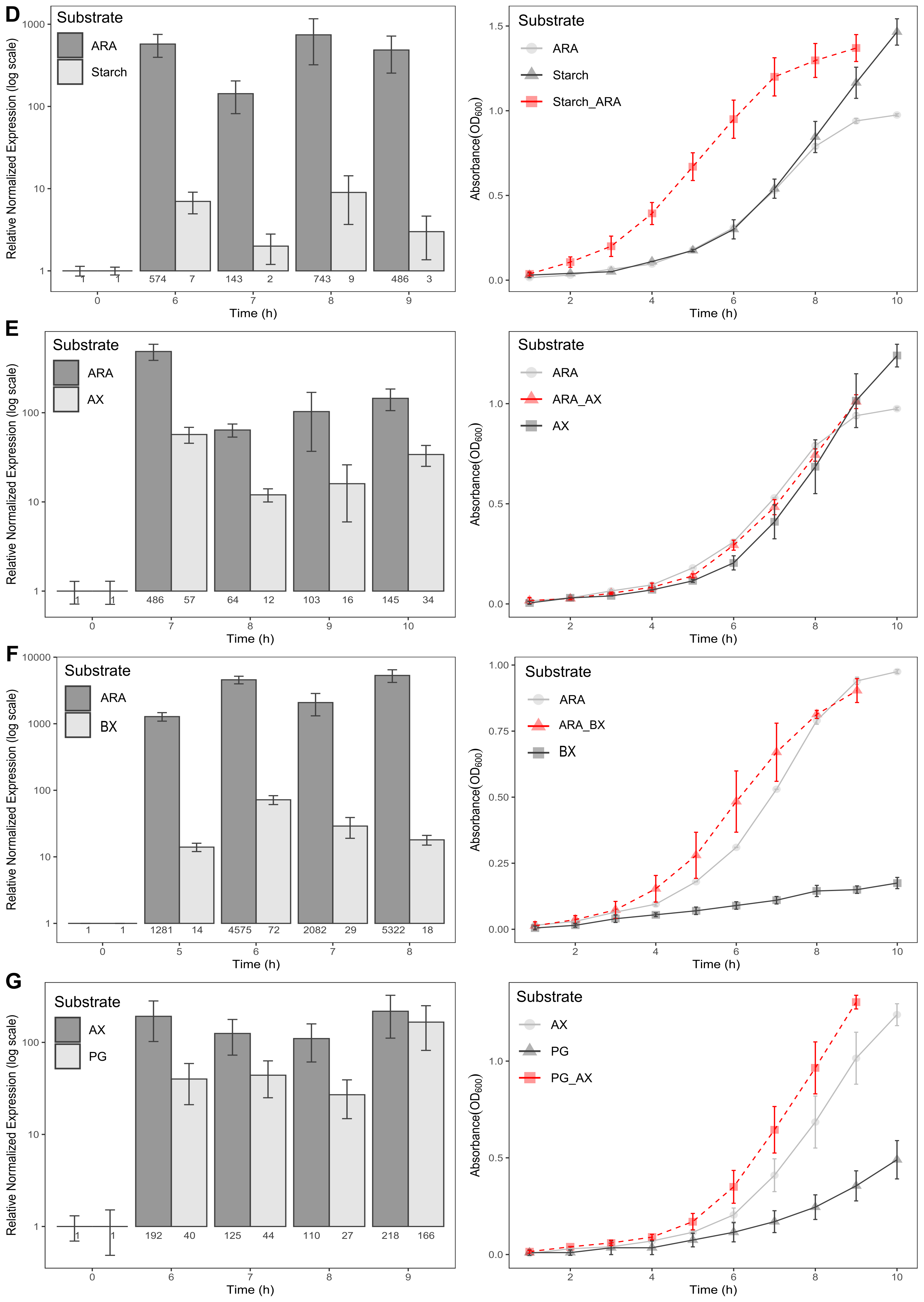
D
Substrate
ARA
Starch
Relative Normalized Expression (log scale)
1000
100
10
1
1 1
574 7
143 2
743 9
486 3
0 6 7 8 9
Time (h)
Substrate
ARA
Starch
Starch_ARA
Absorbance(OD_{600})
1.5
1.0
0.5
0.0
2 4 6 8 10
Time (h)
E
Substrate
ARA
AX
Relative Normalized Expression (log scale)
100
10
1
1 1
486 57
64 12
103 16
145 34
0 7 8 9 10
Time (h)
Substrate
ARA
ARA_AX
AX
Absorbance(OD_{600})
1.0
0.5
0.0
2 4 6 8 10
Time (h)
F
Substrate
ARA
BX
Relative Normalized Expression (log scale)
10000
1000
100
10
1
1 1
1281 14
4575 72
2082 29
5322 18
0 5 6 7 8
Time (h)
Substrate
ARA
ARA_BX
BX
Absorbance(OD_{600})
1.00
0.75
0.50
0.25
0.00
2 4 6 8 10
Time (h)
G
Substrate
AX
PG
Relative Normalized Expression (log scale)
100
10
1
1 1
192 40
125 44
110 27
218 166
0 6 7 8 9
Time (h)
Substrate
AX
PG
PG_AX
Absorbance(OD_{600})
1.0
0.5
0.0
2 4 6 8 10
Time (h)

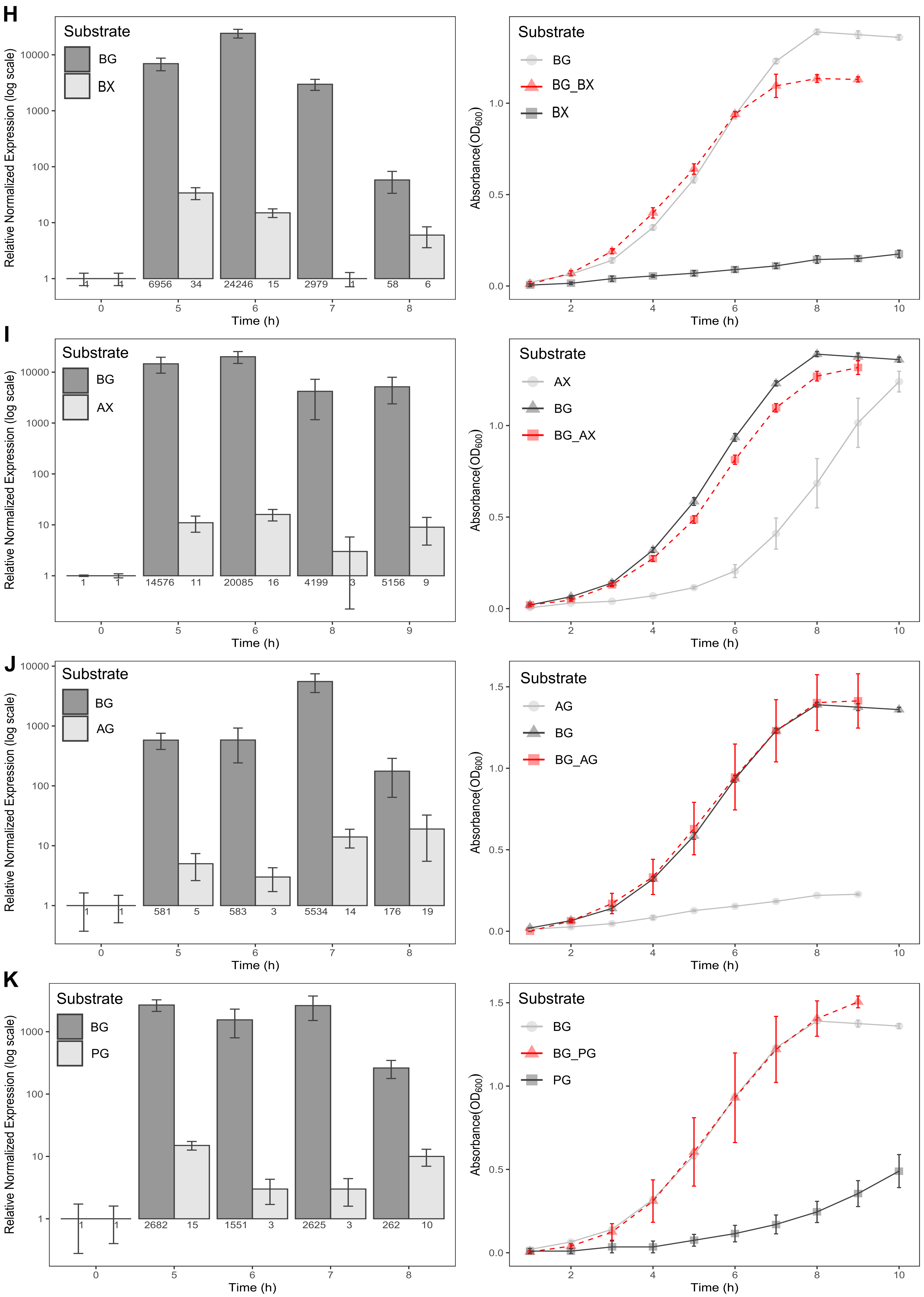
H
Substrate
BG
BX
Relative Normalized Expression (log scale)
1
10
100
1000
10000
1
1
6956
34
24246
15
2979
1
58
6
0
5
6
7
8
Time (h)
Substrate
BG
BG_BX
BX
Absorbance(OD600)
0.0
0.5
1.0
2
4
6
8
10
Time (h)
I
Substrate
BG
AX
Relative Normalized Expression (log scale)
1
10
100
1000
10000
1
1
14576
11
20085
16
4199
3
5156
9
0
5
6
8
9
Time (h)
Substrate
AX
BG
BG_AX
Absorbance(OD600)
0.0
0.5
1.0
2
4
6
8
10
Time (h)
J
Substrate
BG
AG
Relative Normalized Expression (log scale)
1
10
100
1000
10000
1
1
581
5
583
3
5534
14
176
19
0
5
6
7
8
Time (h)
Substrate
AG
BG
BG_AG
Absorbance(OD600)
0.0
0.5
1.0
1.5
2
4
6
8
10
Time (h)
K
Substrate
BG
PG
Relative Normalized Expression (log scale)
1
10
100
1000
1
1
2682
15
1551
3
2625
3
262
10
0
5
6
7
8
Time (h)
Substrate
BG
BG_PG
PG
Absorbance(OD600)
0.0
0.5
1.0
1.5
2
4
6
8
10
Time (h)

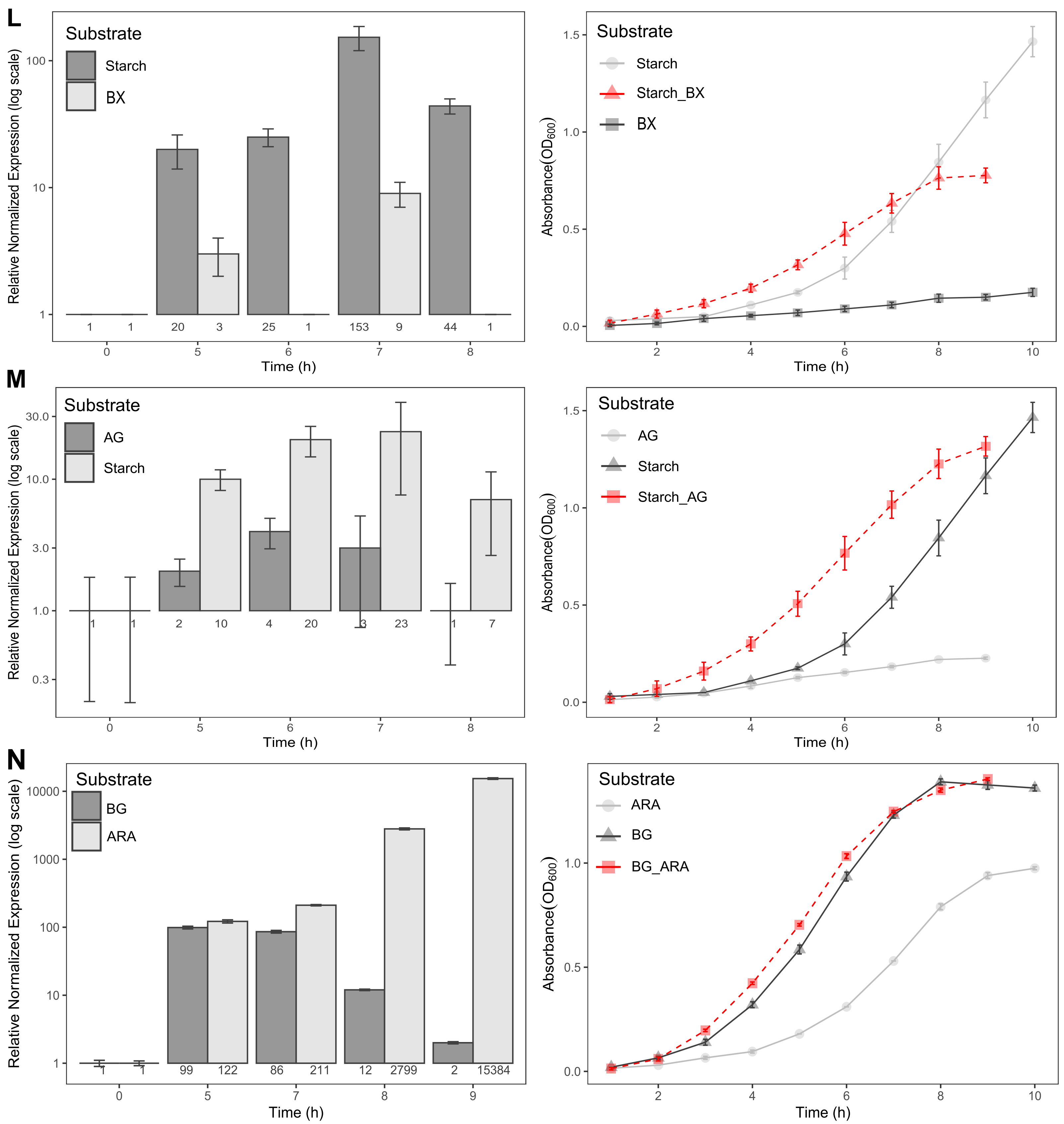

L
Substrate
Starch
BX
Relative Normalized Expression (log scale)
100
10
1
1 1 20 3 25 1 153 9 44 1
0 5 6 7 8
Time (h)
Substrate
Starch
Starch_BX
BX
Absorbance(OD600)
1.5
1.0
0.5
0.0
2 4 6 8 10
Time (h)
M
Substrate
AG
Starch
Relative Normalized Expression (log scale)
30.0
10.0
3.0
1.0
0.3
1 1 2 10 4 20 3 23 1 7
0 5 6 7 8
Time (h)
Substrate
AG
Starch
Starch_AG
Absorbance(OD600)
1.5
1.0
0.5
0.0
2 4 6 8 10
Time (h)
N
Substrate
BG
ARA
Relative Normalized Expression (log scale)
10000
1000
100
10
1
1 1 99 122 86 211 12 2799 2 15384
0 5 7 8 9
Time (h)
Substrate
ARA
BG
BG_ARA
Absorbance(OD600)
1.0
0.5
0.0
2 4 6 8 10
Time (h)
