# Supplementary figures and images for: Transcriptomic analysis of polysaccharide utilization loci reveals substrate preferences in ruminal generalists Segatella bryantii TF1-3 and Xylanibacter ruminicola KHP1
Source: BMC Genomics. 2024 May 20;25:495. doi: 10.1186/s12864-024-10421-z (PMC11107044; doi:10.1186/s12864-024-10421-z)

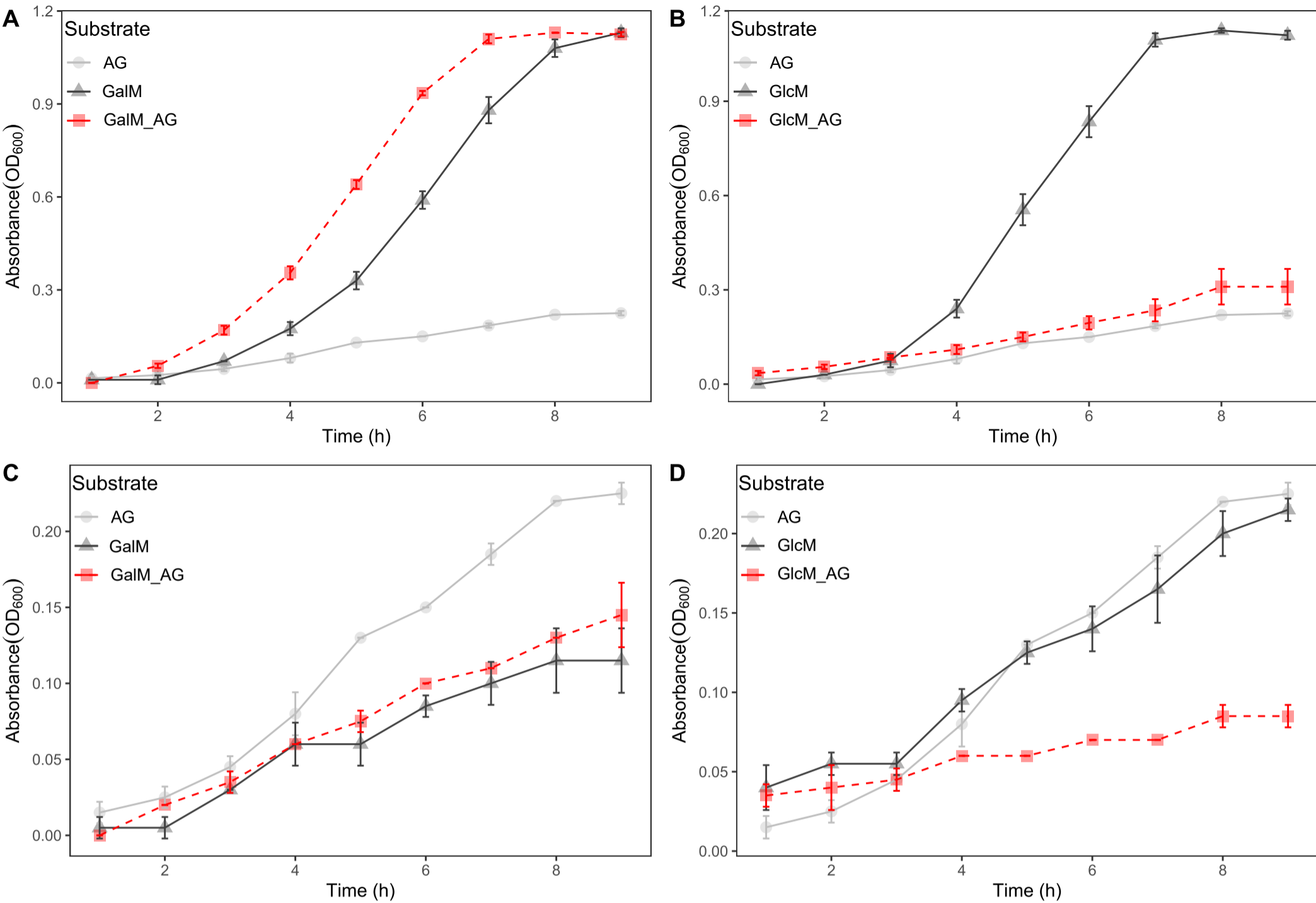

Supplement: Supplementary file 10 — Supplementary Material 10. [file 12864_2024_10421_MOESM10_ESM.pdf]
